# Contraceptive and abortion practices of young Ghanaian women aged 15–24: evidence from a nationally representative survey

**DOI:** 10.1186/s12978-021-01189-6

**Published:** 2021-07-18

**Authors:** Sarah C. Keogh, Easmon Otupiri, Philicia W. Castillo, Naomi W. Li, Joana Apenkwa, Chelsea B. Polis

**Affiliations:** 1 Guttmacher Institute, 125 Maiden Lane, 7th Floor, New York, NY 10038 USA; 2 Kwame Nkrumah University of Science and Technology, Kumasi, Ghana; 3 St. Michael’s Nursing and Midwifery Training College, Pramso, Ghana

**Keywords:** Ghana, Adolescents, Youth, Contraception, Abortion, Reproductive health

## Abstract

**Background:**

Young Ghanaian women experience high rates of unmet need for contraception and unintended pregnancy, and face unique barriers to accessing sexual and reproductive health services. This study provides a comprehensive national analysis of young women’s contraceptive and abortion practices and needs.

**Methods:**

In 2018, we conducted a nationally representative survey of women aged 15–49, including 1039 women aged 15–24. We used descriptive statistics, multivariable logistic and multinomial regression to compare young versus older (25–49 year-old) women’s preferred contraceptive attributes, reasons for discontinuing contraception, quality of counseling, use of Primolut N-tablet, method choice correlates, and friends’ and partners’ influence. We also examined youth’s self-reported abortion incidence, abortion methods, post-abortion care, and barriers to safe abortion.

**Results:**

Among Ghanaian 15–24 year-olds who had ever had sex, one-third (32%) were using contraception. Compared to older women, they had higher desires to avoid pregnancy, lower ever use of contraception, more intermittent sexual activity, and were more likely to report pregnancies as unintended and to have recently ended a pregnancy. Young contraceptors most commonly used condoms (22%), injectables (21%), withdrawal (20%) or implants (20%); and were more likely than older women to use condoms, withdrawal, emergency contraception, and N-tablet. They valued methods for effectiveness (70%), no risk of harming health (31%) nor future fertility (26%), ease of use (20%), and no effect on menstruation (19%). Infrequent sex accounted for over half of youth contraceptive discontinuation. Relative to older women, young women’s social networks were more influential on contraceptive use. The annual self-reported abortion rate among young women was 30 per thousand. Over half of young women used abortion methods obtained from non-formal providers. Among the third of young women who experienced abortion complications, 40% did not access treatment.

**Conclusions:**

Young people’s intermittent sexual activity, desire for methods that do not harm their health, access barriers and provider bias, likely contribute to their greater use of coital-dependent methods. Providers should be equipped to provide confidential, non-discriminatory counseling addressing concerns about infertility, side effects and alternative methods. Use of social networks can be leveraged to educate around issues like safe abortion and correct use of N-tablet.

## Plain English summary

Young Ghanaian women can experience difficulties accessing sexual and reproductive health services, and many are not using contraception despite wanting to avoid pregnancy. To better understand their needs, we describe their preferences and behaviors around contraception and abortion. We surveyed a nationally representative sample of women aged 15–49, and compared young (15–24) versus older (25–49) women’s contraceptive preferences, reasons for stopping contraception, quality of counseling, friends’ and partners’ influence on contraceptive use, and use of abortion. One-third of 15–24 year-olds who ever had sex were using contraception. Compared to older women, young women’s pregnancies were more likely to be unintended and to end in abortion. Young women most commonly used condoms, injectables, withdrawal or implants; and were more likely than older women to use condoms, withdrawal, emergency contraception, and Primolut N-tablet. They preferred methods that were effective, did not harm their health or future fertility, were easy to use, and did not disrupt their menstrual cycle. Over half of young women who stopped contraception did so because they were not having sex regularly. Friends had more influence on contraceptive use among young women than older women. Each year on average, there were 30 abortions per 1000 young women. Over half of young women who had abortions used methods from non-formal providers, and 40% of those who had complications did not get treated. Providers should be equipped to provide confidential, non-discriminatory counseling about contraceptive side effects and options. Social networks can be used to educate women about safe abortion.

## Background

In 2019, young people aged 15–24 comprised 1.2 billion people, or 16% of the world’s population [1]. Young people’s sexual and reproductive health needs are often different from those of their older counterparts. For example, they may have stronger desires to avoid pregnancy, have greater unmet need for contraception, be at higher risk for sexually transmitted infections, and have different contraceptive method preferences and patterns of use [2–4]. In addition, youth may experience stigma from health providers when accessing contraception (particularly if they are unmarried), and particularly in certain low- and middle-income countries, may have to contend with other issues such as early marriage or greater difficulty accessing safe abortion in case of unintended pregnancy [3, 5]. Yet access to comprehensive, confidential and unbiased sexual and reproductive health information and services is a critical part of realizing young people’s sexual and reproductive rights. These fundamental rights are enshrined in various international agreements signed by Ghana, from the broad-ranging 2003 Maputo Protocol [6] to more focused declarations such as the 2016 Outcome Document of the 7th Africa Conference on Sexual Health and Rights [7].

In Ghana, there were nearly 6 million 15–24 year-olds in 2020 [8]. In 2017, an estimated 40% of 15–19 year-old and 88% of 20–24 year-old women had ever had sex [9]. Many Ghanaian women start childbearing early: in 2017, 14% of 15–19 year-olds had had a live birth or were currently pregnant [9]. In 2014,^1^ the majority (58%) of births among 15–19 year-olds were unintended—the highest proportion of all age groups [10]. Unintended pregnancy among Ghanaian adolescents is often highly stigmatized, and can lead to denial by the partner, and shaming by family and health providers [11]. While the proportion of unintended births is comparatively lower (33%) among 20–24 year-olds [10], their perceived risk of unintended pregnancy remains high. For example, in a survey of 250 women aged 18–24 in Accra, 63% felt at risk for future unintended pregnancy [12].

In 2014^1^, 51% of Ghanaian married or sexually-active 15–19 year-olds were not using any contraception despite wanting to avoid a pregnancy—the highest level of unmet need of all age groups [10, 13]. In 2017, just over a quarter (27%) of sexually-active unmarried 15–19 year-olds were using a modern method (predominantly injectables, implants, condoms, emergency contraception and the pill), while 8% were using a traditional method (mostly rhythm and withdrawal), and proportions were even lower for married 15–19 year-olds [9]. Among women aged 20–24, unmet need was somewhat lower (34% among married, 39% among sexually-active unmarried) than in the youngest age group [10], and contraceptive prevalence slightly higher: 28% of married and 39% of sexually-active unmarried women used modern methods, and 5% of married and 10% of sexually-active unmarried women used traditional methods [9].

In Ghana, emergency contraception (EC) is widely used [14–16] and is the most common method among unmarried 20–24 year-olds (currently used by 9%) [9]. While standard EC brands are available in Ghana, recent studies have documented frequent misuse of Primolut N (commonly known as “N-tablet”) as peri-coital contraception, i.e. used immediately before or after sex [17, 18]. Primolut N is a pill containing 5 mg of norethisterone (a synthetic progesterone), intended for use in regulating menstrual cycles, dysmenorrhea, or endometriosis, but there is no evidence that it is effective or safe for use as a contraceptive [18]; for the remainder of the paper, we refer to it as N-tablet. One survey of 85 women aged 15–49 in Kumasi found that 27% had used N-tablet as EC [17].

According to the DHS, the most common reason for non-use of contraception among Ghanaian 15–24 year-olds with unmet need is concern about side effects and health risks [19], and the same was found in a study of 200 women aged 16–19 in Atwima Kwanwoma district [20]. In a cross-sectional survey of 350 18–24 year-olds, 91% of respondents perceived that at least one modern method was unsafe [12]. In-depth interviews with adolescents in Bolgatanga found that women had negative attitudes towards hormonal methods, which they perceived as being linked to infertility [21, 22]. In another qualitative study, myths and misinformation about how hormonal contraception works were common, particularly regarding EC, which respondents feared would disrupt their menstrual cycle and cause long-term side effects [16].

However, other reasons for contraceptive non-use among youth are also at play in Ghana, including cost, lack of confidentiality, negative provider attitudes towards adolescents, and community norms against premarital sex that stigmatize young people’s contraceptive use [23–26]. In a survey of 1203 young people, the most commonly reported barrier to using contraceptive services was embarrassment, along with associated fears about safety and parents finding out [23]. This emphasizes the important role that social networks—be it peers, partners, or parents—may play in young people’s contraceptive decision-making [24].

A portion of unintended pregnancies among 15–24 year-olds will end in abortion. Nationally representative statistics for this age group are lacking, but a few estimates from small-scale studies show widespread use of abortion. A recent study of 15–19 year-old women who had ever had sex in one district found that 58% had been pregnant, 64% of whom had had an induced abortion [27]. Limited evidence suggests abortion, and especially unsafe abortion,^2^ may be particularly prevalent among young women: in a hospital-based survey of 131 women who had an unsafe abortion, most respondents were young, single, and had no children or one child [28]. In Ghana, abortion can be legally obtained in cases of rape, incest, fetal abnormality or disease or ‘defilement of a female idiot’ or to protect physical or mental health. However, many young people are prevented from accessing safe abortion services due to lack of awareness of the law, negative provider attitudes, stigma, shame, distance and cost [29–34]. Among 30 previously pregnant 13–19 year-olds in an Accra slum, 13 of the 15 abortions reported were unsafe [29]. Abortion methods used by young Ghanaians include misoprostol (alone or in combinations with other drugs) [30, 31], inserting herbs into the vagina or drinking concoctions [26].

The government of Ghana has implemented several national initiatives that promote a human rights-based approach to adolescent sexual and reproductive health  programming [35, 36]. The Ministry of Health/Ghana Health Service has developed policies, protocols and standards that seek to improve the quality of, and access to youth-friendly sexual and reproductive health information and services. Ghana has also put a strong emphasis on contraceptive services uptake to meet its 2017 pledge (at the London Family Planning Conference) to increase contraceptive prevalence among sexually active adolescents by 3% by 2020. In addition, the Ministry of Gender, Children and Social Protection (which coordinates the efforts of government ministries, agencies, the private sector, and non-governmental organizations to ensure the welfare of women and girls) developed a 5-year strategic plan (2018–2022) designed to address adolescent pregnancy in Ghana [35].

Meeting the government’s targets for improving young people’s sexual and reproductive health and rights requires a better understanding of their needs and behaviors regarding contraception and abortion. The studies summarized above provide crucial illustrations of the numerous challenges faced by Ghanaian youth, but few are nationally representative. National policymaking requires an understanding of needs for Ghana as a whole, to complement small-scale in-depth studies and help assess their generalizability to the rest of the country. This study aims to help fill this gap by examining the contraceptive and abortion practices and needs of a nationally representative sample of young women aged 15 to 24. We investigate understudied aspects of contraceptive practices in this age group, such as attributes important to young people, reasons for discontinuing contraceptive use, quality of counseling, N-tablet use and beliefs, factors associated with choice of more or less effective methods, and the influence of friends and partners on contraceptive use. We also examine self-reported abortion practices in greater depth than previous studies, looking at national incidence, abortion methods, post-abortion care, and barriers to accessing safe abortion. Throughout, we compare young women’s contraceptive and abortion practices with those of their older counterparts, thus further illuminating the particularities of young people’s behaviors and their specific needs in relation to the rest of the population.

## Methods

### Data collection

Data for this analysis come from a 2018 nationally representative household-based survey of Ghanaian women aged 15–49. Information on the study design and sampling plan are described in depth elsewhere [37]. Trained interviewers administered a structured questionnaire face-to-face in a private area using password-protected Android tablets enabled with Open Data Kit. Informed consent was obtained from all adult women, and parental or guardian (or husband) consent and minor assent were obtained for women under 18. This study received ethical approval from the Kwame Nkrumah University of Science and Technology (KNUST) Committee on Human Research, Publication and Ethics (CHRPE/AP/210/18), the Johns Hopkins Bloomberg School of Public Health (00008463) and the Guttmacher Institute’s Institutional Review Board (IRB00002197).

KNUST conducted fieldwork, with technical support from the Guttmacher Institute and the Performance Monitoring and Accountability 2020 team at the Johns Hopkins Bloomberg School of Public Health. Interviews were conducted in English or in the respondent’s local language. Since there is an abundance of unwritten languages spoken in Ghana, interviewer training included establishing agreement on translation of the survey questions. All participating households received a bar of soap as compensation for their time. The survey response rate was 99.3%, giving us a final sample of 4722 women aged 15–49. The study funders had no role in study design, data collection, analysis, interpretation of the data, or in the writing and decision to submit the manuscript.

### Data analysis

Our analysis sample included all respondents who had ever had sex (N = 4139, 88% of total sample), of whom 1039 were aged 15–24 (66% of total 15–24 year-old sample). We performed all data cleaning and analyses in Stata version 16 (StataCorp 2019) using *svy* commands to account for complex sampling design with the *subpop* option to restrict our analytic population. All estimates presented (proportions, odds ratios, risk ratios) are weighted and all Ns are unweighted. We stratified analyses by age group to enable comparison of contraceptive and abortion practices between young people (aged 15–24) and their older counterparts (aged 25–49). Results focus on patterns among 15–24 year-olds, and we note key differences by age where relevant.

### Contraceptive use measures and method classification

We collected four measures of contraceptive use: ever use and current use, and among current non-users, recent use in the past two years and intended use. The analysis mainly focuses on current and ever use, except in examining recent use in the context of discontinuation and intended use in the context of method attributes. When asking respondents about their contraceptive use, we probed about each specific method (listed in Table 1) to ensure they did not accidentally omit any method. In addition, because we had a particular interest in N-tablet, we included questions on its ever use, reasons for use, and beliefs about its effectiveness.

**Table 1:** Percent of respondents currently using each method, among 15–24 year-olds and 25–49 year-olds who ever had sex

	Among total sample			Among current contraceptive users		
	15–24 (N = 1039)	25–49 (N = 3100)	p-val	15–24 (N = 330)	25–49 (N = 946)	p-val
*Any method*	31.9	30.4	0.00	NA	NA	NA
Female sterilization (tubal ligation)^a^	0.0	1.4	0.00	0.0	4.7	0.00
Hormonal/LARC methods requiring clinic visit						
Implant	6.3	7.2	0.39	19.8	23.8	0.19
IUD	0.3	0.9	0.28	0.9	3.0	0.25
Injectable (3-month)	6.2	6.4	0.87	19.4	20.9	0.63
Injectable (1-month)	0.4	0.8	0.36	1.4	2.5	0.31
Hormonal methods not requiring clinic visit						
Pill	4.4	4.4	0.98	13.8	14.5	0.76
Emergency contraception	4.9	1.8	0.00	15.5	5.8	0.00
Condoms^b^	7.0	2.4	0.00	22.1	7.9	0.00
Modern FABMs^c^						
LAM	0.2	0.4	0.55	0.8	1.2	0.51
Standard days Method/CycleBeads	1.6	2.0	0.54	5.0	6.4	0.44
Traditional methods						
Rhythm	4.7	3.9	0.30	14.9	12.8	0.43
Withdrawal	6.5	2.9	0.00	20.4	9.6	0.00
Washing	0.9	0.7	0.59	2.8	2.3	0.65
Other traditional	0.4	1.2	0.13	1.3	4.0	0.11
N-tablet^d^	1.7	0.8	0.02	5.3	2.6	0.02

All Ns are unweighted. Method prevalence among contraceptive users can add up to > 100% because women can use multiple methods

^a^Male sterilization was not reported by any respondent

^b^Includes female condom users (N = 5 current total users)

^c^FABM: fertility awareness-based method

^d^“N-tablet” or Primolut N is a pill containing 5 mg of synthetic progesterone, intended for use in regulating menstrual cycles, dysmenorrhea, or endometriosis. In Ghana, N-tablet is sometimes misused as contraception or emergency contraception

We classified contraceptive methods into two categories. “Hormonal and long-acting reversible contraceptive (LARC) methods and sterilization” (the methods considered most effective in preventing pregnancy) included implants, injectables (1- or 3-month), IUDs (the majority of which are non-hormonal in Ghana [38]), oral contraceptive pills, EC and tubal ligation.^3^ “Other methods” (methods that are considered less effective or lack scientific evidence of effectiveness) comprised condoms, diaphragms, modern fertility awareness-based methods (FABMs; including lactational amenorrhea method and Standard Days Method/CycleBeads), traditional methods (rhythm, withdrawal, washing and folkloric methods), and N-tablet.

### Contraceptive method attribute analysis

We asked current and intended contraceptive users about the attributes most important to them when choosing their current (most effective) method or their future method. Responses were prompted (with multiple responses allowed) following 17 pre-coded contraceptive attributes (listed in Table 2). Utilizing classifications from previous studies [39, 40], we grouped these attributes into four categories: (1) effectiveness at preventing pregnancy; (2) concerns around health risks and side effects; (3) practicality (e.g. ease of use, duration of protection, accessibility, frequency of administration); and (4) social/normative (provider recommendation and partner approval). We analyzed attributes separately for current and intended users. We confirmed no significant difference in the mean number of attributes cited by younger (15–24) and older (24–49) participants (2.5 vs. 2.6), enabling attribute prevalence comparisons by age.

**Table 2:** Percent of respondents aged 15–24 citing each attribute as a reason for choosing their current or intended method

	All current users^a^	All intended users^b^
	N = 300	N = 394
*Effectiveness*	*70*	*69*
Most effective at preventing pregnancy	70	69
*Health risks and side effects*	*56*	*58*
No risk of harming health	31	37
No risk of harming future fertility	26	29
No effect on regular monthly bleeding	19	20
Lighter or no bleeding	3	5
No unpleasant side effects (other than changes in bleeding)	7	13
For its other health benefits (regulating cycle, acne prevention)	1	1
Doesn't interfere with sexual pleasure	6	3
*Practicality*	*44*	*43*
Easy to use	20	24
Don't need to remember to use it	9	10
Can be used for long time without need for re-supply	11	8
Can use it without anyone knowing	16	15
Easy to obtain	12	14
Cheap/no cost	5	5
Only one available at facility	0	0
*Social/normative*	*13*	*7*
Health provider recommended it	3	2
Partner/husband approved	12	5
*Mean total number of attributes*	2.5	2.6

All Ns are unweighted. Responses are in relation to current most effective method or future preferred method, and respondents were prompted about each attribute

^a^Excludes women who are pregnant, trying to get pregnant or sterilization users

^b^Current non-users who intend to use a method in future

### Contraceptive discontinuation and quality of counseling

We explored reasons for contraceptive discontinuation in the last two years, classifying responses into 26 pre-coded reasons that we then reduced to 21 categories through the combination of similar reasons and exclusion of reasons lacking responses (see Table 3). We compared various aspects of quality of contraceptive counseling reported by current and recent users of hormonal methods, analyzing differences between current and recent users within age groups, as well as differences between age groups overall.

**Table 3:** Contraceptive discontinuation: prevalence and reasons among 15–24 and 25–49 year-olds

	15–24	25–49	
	N = 463	N = 1260	p-value
% of recent users^a^ who discontinued their method	28.8	25.4	0.23
*Reason for discontinuing*	N = 133	N = 314	
Became pregnant while using (method failure)	8.2	8.0	0.95
Wanted to become pregnant	9.8	22.9	0.01
Up to god/fatalistic	0.7	0.2	0.44
Wanted a more effective method	0.0	2.7	0.16
No partner/not having sex	27.7	18.0	0.14
Infrequent sex^b^	26.3	14.8	0.02
Menopausal/hysterectomy	0.0	0.4	0.51
Did not think she could get pregnant	0.0	3.4	0.07
Health concerns	8.5	11.7	0.40
Fear of infertility	1.9	2.5	0.71
Changes to monthly bleeding	11.0	13.8	0.45
Other side effects (other than changes in bleeding)	4.5	5.5	0.71
Interferes with body's processes	6.2	6.5	0.93
Interferes with sexual pleasure	3.2	0.8	0.03
Decreased interest in sex	1.0	0.0	0.19
Inconvenient to use	1.8	2.5	0.70
Partner/husband opposed	2.0	3.1	0.47
Lack of access/too far	0.5	0.2	0.00
Costs too much	0.8	1.4	0.65
Preferred method was not available/method not available	1.3	1.2	0.93
Other	13.9	9.9	0.31

^a^Respondents who used a method within the past 2 years

^b^Includes "partner away for multiple days"

### Multivariable model predicting current contraceptive use

Using a multivariable multinomial model, we examined independent correlates of using hormonal/LARC methods or tubal ligation (vs. other methods) or no method (vs. other methods). We selected “other methods” as the reference category because we deemed it more informative to compare hormonal/LARC methods to “other methods” rather than to “no method”. Covariates included ecological zone (Northern, Middle, Coastal), residence (urban, rural), union status (in union versus not), educational attainment (none, primary, middle or higher), wealth (poorest 60%, richest 40%), time since last sex (one month or less, over a month), pregnancy and birth history (never pregnant, ever pregnant with no birth, one birth, two or more births), and the importance of avoiding pregnancy (very important, somewhat important, not at all important). We selected these covariates based on their association with contraceptive use and abortion in Ghana [10, 41–46]. The model excludes women who are pregnant or trying to get pregnant.

### Social network analyses

We examined the influence of friends and partners on respondents’ contraceptive decision-making. First, we used descriptive statistics to examine patterns of decision-making, discussion of, and opposition to contraception. We then conducted multivariable logistic regression to assess the association between social network support or opposition to contraception and respondents’ current contraceptive use. Independent variables included the respondent’s view on contraception (supports or opposes), the main contraceptive decision-maker (respondent, partner, joint, other), the partner’s view on contraception (opposes, supports, doesn’t know), and the proportion of the respondent’s friends who oppose contraception (most, half, few, none). We conducted an additional multivariable logistic regression to assess associations between partners’ and friends’ opposition to contraception and respondent opposition. Both models are adjusted for background characteristics (ecological zone, residence, union status, educational attainment and wealth).

### Abortion measures and methods

We calculated the prevalence of self-reported abortion within the past three years among women who ever had sex. Abortion included respondents’ direct reports of successfully “ending a pregnancy” as well as reports of taking action to successfully “bring back late menses” if the respondent believed she was pregnant at the time. From this, we computed a 3-year annualized abortion incidence rate per 1000 women.^4^ Due to the small sample of 15–24 year-olds who had an abortion, we did not look at correlates of abortion.

We calculated the prevalence of different types of abortion methods used: surgical abortions by a formal provider,^5^ mifepristone and/or misoprostol, unknown pills from a formal provider, unknown pills from a non-formal provider,^6^ N-tablet, and “other”.

### Sensitivity analysis

To determine how sensitive our findings were to focusing on 15–24 year-olds versus focusing more narrowly on 15–19 year-olds, we conducted a sensitivity analysis repeating all tabulations and comparing 15–19 year-olds to 20–49 year-olds. The broad findings and conclusions were the same for both analyses, so we do not present these in the results section.

## Results

### Sample description

Compared to 25–49 year-olds, 15–24 year-olds who ever had sex were significantly more likely to be rural, in the poorest 60% of households in the sample, never married nor cohabitating, and with middle school or higher education (Appendix A). They were less likely to have had sex in the past month, to have had two or more births, to be trying to become pregnant, and they were more likely to find it very important to avoid pregnancy. Yet they were also more likely to have never used contraception and to classify their current pregnancy or last birth as unintended.

### Patterns of contraceptive use

Among the 32% of young women who were current contraceptive users, 41% were using methods that required a clinic visit to obtain—predominantly 1- or 3-month injectables (21%) and the implant (20%) (Table 1). A sizeable proportion were using a hormonal method that did not require a clinic visit, namely the pill (14%) or EC (16%), while 22% used condoms and 6% used a modern FABM (primarily Standard Days/CycleBeads). The main traditional methods used were withdrawal (20%) and rhythm (15%), while 5% used N-tablet. Compared to their older counterparts, young women were less likely to use female sterilization, but more likely to use EC, condoms, N-tablet and withdrawal.

**Fig. 1:**
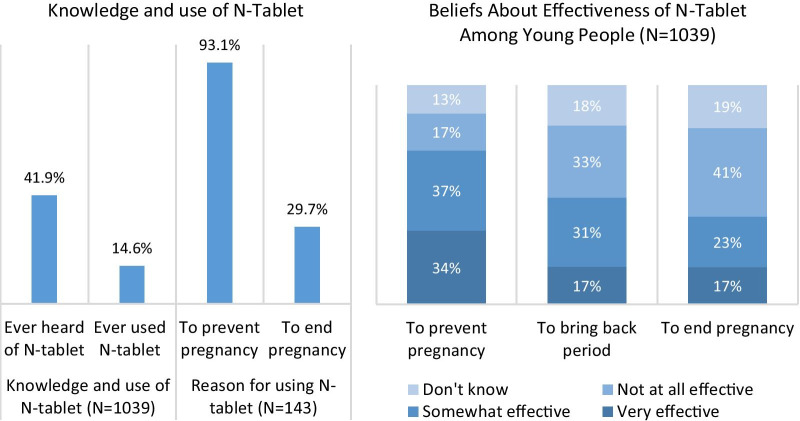
Use of and beliefs about N-tablet among 15–24 year-old Ghanaian women

Less than half (42%) of young women had heard of N-tablet and 15% had ever used it, of whom 93% used it to prevent pregnancy and 30% to try to end a pregnancy (Fig. 1). Over a third of 15–24 year-olds considered N-tablet to be very effective at preventing pregnancy, but fewer considered N-tablet very effective for bringing back a late period or ending a pregnancy (17% for each); beliefs among women 25 or older were similar.

When choosing a contraceptive method, young current contraceptive users ranked categories of important attributes in the following order: effectiveness at preventing pregnancy (mentioned by 70%), no health risks or side effects (56%), practicality (44%), and social or normative factors (13%) (Table 2). The most frequently cited health-related attributes were that the method have no risks of harming health (31%) or future fertility (26%), and no effect on menstruation (19%). The most frequently cited practicality attributes were ease of use (20%) and possibility of covert use (16%), while the most cited social/normative attribute was partner approval (12%). The preferred attributes for contraceptive non-users who intended to use a method followed a similar pattern. There were no significant differences in cited method attributes between 15–24 and 25–49 year-olds.

Around 29% of young women who had used a method in the past two years had discontinued use and were not using any other method by the time of the survey (Table 3). When asked about their reason for discontinuing, young women most commonly cited having no sexual partner (28%), infrequent sex (26%), other unspecified reasons (14%), and changes to monthly bleeding patterns (11%). Compared to their older counterparts, they were less likely to have discontinued because they wanted to become pregnant, and more likely to have discontinued because of infrequent sex, because their method interfered with sexual pleasure, or because of difficult access.

### Correlates of contraceptive use

Women aged 15–24 were more likely to be using no method (vs. “other methods”) if they had sex over 1 month ago (RRR = 5.15, 95% CI: 2.60–10.21), were living in a rural area (RRR = 2.31, 95% CI: 1.06–5.04), or had two or more births (RRR = 2.49, 95% CI: 1.01–6.14) (Table 4). Their likelihood of using hormonal/LARC methods (versus other methods) significantly increased with their number of pregnancies and live births.

**Table 4:** Adjusted multinomial relative risk ratios (RRR) of using no method and using hormonal/LARC methods, relative to using other methods, by sociodemographic characteristics among 15–24 year-olds

	Relative to using other methods	
	No method	Hormonal/LARC methods^a^ and sterilization
	RRR (95% CI)	RRR (95% CI)
*Zone*		
Northern	1.90 (0.80, 4.50)	1.90 (0.87, 4.19)
Middle	(ref)	(ref)
Coastal	1.08 (0.51, 2.29)	0.96 (0.50, 1.85)
*Residence*		
Urban	(ref)	(ref)
Rural	2.31 (1.06, 5.04)*	1.43 (0.69, 2.98)
*Union status*		
Not in union	(ref)	(ref)
In union (married/cohabiting)	0.65 (0.31, 1.38)	0.96 (0.45, 2.02)
*Sexual activity*		
Had sex in last month	(ref)	(ref)
Had sex > 1 month ago	5.15 (2.60, 10.21)*	0.71 (0.39, 1.29)
*Pregnancy/birth history*		
Never pregnant	(ref)	(ref)
Ever pregnant, no birth	1.34 (0.53, 3.37)	2.81 (1.03, 7.66)*
1 birth	1.10 (0.52, 2.31)	3.01 (1.58, 5.77)*
2 + births	2.49 (1.01, 6.14)*	4.62 (1.95, 10.94)*
*Education*		
None	(ref)	(ref)
Primary	0.15 (0.01, 1.70)	0.29 (0.02, 3.77)
Middle and higher	0.22 (0.02, 1.99)	0.41 (0.04, 4.76)
*Wealth*		
Poorest 60%	(ref)	(ref)
Richest 40%	1.88 (0.92, 3.82)	1.37 (0.76, 2.45)
*Importance of avoiding pregnancy*		
Very important	(ref)	(ref)
Somewhat important	1.46 (0.47, 4.54)	0.95 (0.35, 2.56)
Not at all important	1.36 (0.45, 4.09)	0.40 (0.16, 0.99)
*Number of women*	167	207

Model excludes women who are pregnant or trying to get pregnant

^a^Hormonal/LARC methods include implant, IUD, injectable (3-month and 1-month), pill and EC

^*^p < 0.05

Although the following differences did not reach statistical significance, compared to their poorer counterparts, wealthier young women had slightly higher relative risks of using hormonal/LARC methods, or of using no method, versus using other methods (including traditional methods and FABMs). In contrast, among 25–49 year-olds, wealthier women were slightly less likely to use no method (RRR = 0.66, 95% CI: 0.42–1.04) or hormonal/LARC methods (RRR = 0.52, 95% CI: 0.34–0.80) compared to other methods (data not shown), similar to findings from a previous analysis of these data suggesting use of FABMs was higher among older, wealthier, more educated Ghanaian women [47]. The opposite pattern among young women illustrates the complex relationship between wealth and traditional method use observed in other studies [48]. Other patterns of association in the 25–49 age group were in the same direction as those among 15–24 year olds.

Over half (56%) of 15–24 year-olds who ever had sex had discussed contraception with their partner (Table 5). The majority of young women (73%) reported being the main decision-maker regarding contraceptive use, although 19% reported deciding jointly with their partner, and 6% said that their partner was the main decision-maker. Over a quarter (27%) of young women opposed contraception, and 28% said that their partner opposed it. When asked why their partner opposed it, 52% of respondents mentioned health concerns, 35% cited moral reasons, and 25% said their partner did not see a need for it.

**Table 5:** Role of social networks in contraceptive decision-making among 15–24 and 25–49 year-olds

	15–24	25–49	p-value
	N = 1039	N = 3100	
Respondent opposes contraception	27.0	26.9	0.52
*Role of partner*			
Ever discussed contraception with partner	55.8	58.9	0.41
Decision-maker regarding contraceptive use			0.80
Mainly respondent (with or without provider)	72.5	73.1	
Mainly partner	5.8	5.0	
Joint	19.2	19.5	
Other	2.4	2.4	
Partner opposes contraception	27.8	34.5	0.00
Why?			
Does not see need for it (up to god, wants child, infrequent sex)	24.7	37.0	0.00
Moral reasons^a^	34.5	34.4	0.98
Health concerns (side effects, fertility impairment)	51.5	49.8	0.67
Accessibility issues (cost, access)	1.8	1.0	0.21
Family opposes	3.6	3.5	0.97
Interferes with pleasure	1.5	1.0	0.52
Other	12.1	7.4	0.05
*Role of friends*			
Ever discussed contraception with friends	58.3	59.1	0.85
Of these, % who ever discussed side effects with friends	72.8	76.9	0.13
Of these, % who were discouraged from using certain methods by these discussions	68.2	57.1	0.00
Proportion of friends who oppose contraception			0.10
Most	23.1	25.8	
About half	13.2	13.9	
Few	45.4	45.8	
None	18.3	14.6	

^a^Believes it is women's duty to have children, his religion opposes it, or associates it with promiscuity

Over half of 15–24 year olds (58%) had talked with friends about contraception, of whom 73% had discussed side effects. Among those who discussed side effects, 68% said that these discussions had discouraged them from using certain contraceptive methods. While the majority of young women (64%) reported that few or none of their friends opposed contraception, nearly a quarter (23%) said that “most of their friends” opposed it.

There were significant differences between younger and older women in the prominence of social networks in contraceptive decision-making. Compared to their older counterparts, 15–24 year-olds were more likely to have been put off from using certain methods by conversations with friends (68% vs. 57%, p < 0.01). Younger women were also less likely to have a partner who opposes contraception (28% vs. 35%, p < 0.01), and if he did, he was less likely to oppose it because he did not see the need (25% vs. 37%, p < 0.01) and more likely to oppose it for other (unspecified) reasons (12% vs. 7%, p = 0.05).

In a logistic regression adjusted for background characteristics, young women were more likely to be using contraception if contraceptive decisions were made jointly (OR = 87.87, 95% CI: 26.46–291.77) or by the partner alone (OR = 4.22, 95% CI: 2.29, 7.77) rather than by the respondent (Appendix B). They were less likely to be using contraception if they personally opposed contraception (OR = 0.37, 95% CI: 0.16–0.84) or if their partner opposed contraception (OR = 0.49, 95% CI: 0.25–0.98), but friends' opinions on contraception were not significantly associated with respondents’ contraceptive use. In the regression on respondent opinions (Appendix C), women were more likely to oppose contraception if their partner opposed it (OR = 13.17, 95% CI: 8.16–21.23) or if they did not know his opinion (OR = 6.75, 95% CI: 3.82–11.94), and if most of their friends opposed it (OR = 1.89, 95% CI: 1.23–2.91). Of note, while friends’ opinions were not independently associated with respondents’ contraceptive use once we controlled for respondents’ opinions, partner’s opinion continued to be independently associated with contraceptive use after controlling for respondent opinion (Appendix B)).

**Fig. 2:**
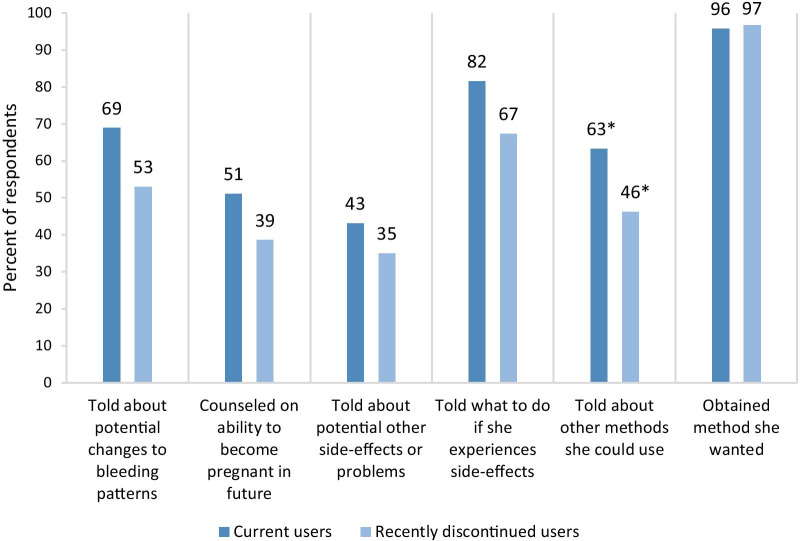
Quality of contraceptive counseling received by 15–24 year-olds. *Difference between current and recently discontinued users is significant (p < 0.05)

The quality of recent contraceptive counseling may also affect contraceptive use or discontinuation. Compared to current users, young women who had recently discontinued were less likely to have been counseled on other methods they could use (46% vs. 63%, p = 0.02) (Fig. 2). Other indicators for quality of counseling were non-significantly worse for those who discontinued (potentially due to small sample sizes); similar patterns were significant among 25–49 year-olds. Older women were more likely to have been told about other methods they could use compared to their younger counterparts (72% vs. 58%, p < 0.01; data not shown).

### Abortion practices

Seven percent of young women who ever had sex reported ending a pregnancy, and 6% reported doing something to bring back late menses, in the last three years (Table**6**). These proportions were significantly higher than among 25–49 year-olds (5%, p = 0.03 and 3%, p < 0.01, respectively). However, the annualized population incidence rate for self-reported abortions of 30 per thousand was the same for both age groups, since young women’s higher probability of reporting an abortion was counterbalanced by their lower probability of having ever had sex.

**Table 6:** Abortion and post-abortion care among 15–24 and 25–49 year-olds

	**15–24**	**25–49**	
	N = 1039	N = 3100	p-value
**Abortion estimates**			
% who successfully ended a pregnancy within last 3 years	7.0	4.8	0.03
% who successfully "brought back late menses" within last 3 years	5.6	2.6	0.00
Overall abortion incidence (abortions per 1000 women) ^a^	29.5	29.9	0.76
**Among respondents who had an abortion**	N = 116	N = 200	
Method used			
Pills: mifepristone and/or misoprostol	21.8	24.3	0.71
Pills: unknown but obtained from a formal provider ^b^	14.3	12.3	0.61
Pills: unknown and obtained from a non- formal provider	14.7	16.9	0.62
Surgical procedure by formal provider	17.0	16.3	0.90
N-tablet	3.2	5.5	0.37
Other ^c^	42.6	43.3	0.92
% who had complications	32.4	34.5	0.70
Of those with complications, % who received treatment	59.9	57.3	0.81
In facility/with formal health provider	39.7	45.1	0.62
At traditional healer/birth attendant	3.7	2.6	0.72
At pharmacy	17.0	14.0	0.68
Other	0.0	1.3	0.41
Contraceptive use at time of pregnancy			
Yes, modern method^d^	25.0	25.7	0.91
Yes, traditional method^e^	12.7	15.8	0.63
No	65.1	61.1	0.60

All Ns are unweighted

^a^Incidence is out of all women (including those who never had sex)

^b^Formal providers comprise private doctors, midwives, hospitals, clinics (including mobile and family planning clinics), pharmacies, NGOs, CHPS and maternity homes

^c^Examples of other: Traditional remedy, inserted or injected something, procedure from traditional healer/birth attendant

^d^Modern methods: IUD, implant, injectables, EC, pill, Standard Days/beads, LAM

^e^Traditional methods: rhythm, withdrawal, washing, other traditional

Young women used a variety of methods to end their pregnancies. They most commonly reported using some “other” method (43%), which includes traditional remedies, inserting or injecting something, or visiting a traditional healer. The second most common method was mifepristone and/or misoprostol pills (22%), followed by a surgical procedure with a formal provider (17%), unknown pills from a non-formal provider (15%), unknown pills from a formal provider (14%), and N-tablet (3%). Of the 28 young women who accessed their method outside of a health facility, 19 knew that certain facilities offered safe abortion services. Of those women, six chose not to go to a health facility because it was too costly, four chose not to go because of concerns about confidentiality, and three did not go because it was their partner’s decision (data not shown).

Nearly one-third (32%) of respondents who ended their pregnancy experienced complications, 60% of whom received treatment for them: 40% from a formal provider, 17% at a pharmacy, and 4% from a traditional healer.

A quarter of young women who had an abortion had been using a modern contraceptive method at the time of pregnancy, 13% had been using a traditional method, while the majority (65%) had not been using contraception.

## Discussion

Our analysis has highlighted several practices particular to young people in Ghana, relating specifically to 1) abortion, 2) contraceptive use patterns, methods, and preferred attributes, 3) use of N-tablet, and 4) social network influence. We discuss key findings for each domain.

### Abortion

Compared to their older counterparts, 15–24 year-olds had higher desires to avoid pregnancy, lower ever use of contraception, a greater propensity to report recent pregnancies as unintended, and were more likely to report ending a pregnancy. This lends support to earlier evidence suggesting that abortions are more common among young women [28]. The estimated self-reported abortion incidence rate of 30 abortions per thousand young women is likely an underestimate due to widespread reluctance to report this highly stigmatized practice, although asking about actions to bring back late menses considerably increased the self-report estimate [45]. Young people reported inadequate access to safe abortion services, due mainly to insufficient knowledge, cost, and worries about confidentiality. Previous studies have documented similar barriers, including poor knowledge of the law, negative provider attitudes, cost, stigma and distance [29–34]. These barriers push young people to use potentially unsafe methods to end their pregnancies: over half of young women reported using methods not obtained from formal providers. An additional 22% of women self-induced using mifepristone and/or misoprostol pills (possibly more, since another 29% were unsure which pills they had obtained). If accompanied with adequate counseling on correct use, medication abortion provides a safe and affordable option for young people. However, inaccurate counseling by informal providers may put women at further risk of complications. A third of young women experienced complications from their abortion, and it is likely that some of the 40% who did not access treatment were prevented from doing so due to distance, cost, confidentiality concerns, inadequate knowledge, and other barriers. There is an urgent need to disseminate accurate information about safe abortion and post-abortion care, and to train providers to ensure they are willing and able to provide confidential abortion counseling and services to young people.

Among young women who reported having an abortion, only a quarter had been using a modern contraceptive method at the time of pregnancy – and this is among a subset of young people highly motivated to avoid a birth. Contraceptive use may be even lower among women with less motivation or fewer means to prevent a birth. For example, in-depth interviews with 30 previously pregnant 13–19 year-olds in an Accra slum found that none had been using contraception prior to pregnancy [29]. These findings show a clear need for more effective pregnancy prevention strategies tailored to young Ghanaian women.

### Contraceptive use patterns, methods, and preferred attributes

Young people’s significantly higher use of condoms, withdrawal, EC and N-tablet (as compared to older women) suggests they tend to use coital-dependent methods accessible without a clinic visit, which generally also happen to be less effective at pregnancy prevention. These types of methods may be better suited to their more intermittent sexual activity. The fact that not being in union and infrequent sex were top reasons for discontinuing contraception suggests a potential perceived unsuitability of certain long-acting methods to young people’s needs. In in-depth interviews with 32 unmarried sexually active 18–24 year-olds in Accra, women described how it was often difficult to plan sexual encounters, and for this reason most of them used EC, often as back-up if traditional methods (usually withdrawal) failed [16]. In these circumstances, pericoital methods that can be used immediately before, during or after sex may be particularly appealing. However, other reasons are also contributing to young people’s choice of short-acting over longer-acting methods. For example, 15–24 year-olds were significantly more likely than their older counterparts to discontinue a method because of difficult access. Methods that require a clinic visit are more difficult to access, given distance and cost [23], as well as provider bias and confidentiality concerns [23–26]. In our study, according to women’s reports, providers were significantly less likely to counsel younger women on the range of contraceptive options available to them, a bias that may hinder young women from accessing the method that suits them best; indeed, lack of counseling on other options was associated with subsequent discontinuation.

Young people’s preference for condoms and withdrawal over hormonal methods appears somewhat discrepant with their ranking effectiveness as their most valued method attribute. One explanation is inaccurate perceptions around the relative effectiveness of various contraceptive methods [37]. Alternatively, their method choices may be explained by other top attributes: no risk of harming health or future fertility. Because young women are less likely to have been pregnant, they have yet to demonstrate their fecundity in a society that puts a high value on childbearing capabilities [49]. Given the common misperception that hormonal methods may lead to infertility [21, 22], young women may avoid methods that they fear might damage their future fertility. In fact, young women with more pregnancies were increasingly likely to be using hormonal/LARC methods rather than other methods, perhaps because they did not worry as much about the effect of these methods on their future fertility, and were more concerned about method effectiveness at avoiding additional pregnancies. Condoms and withdrawal also possess another key attribute valued by young people: no effect on menstruation. While other studies suggest that young Ghanaians do sometimes associate EC with menstrual disruptions and health risks, it is not believed to affect future fertility [16]. This, coupled with ease of use, no need for a clinic visit, and ability to use pericoitally, may help explain EC’s high prevalence among young Ghanaian women.

### Use of N-tablet

The use of N-tablet as a contraceptive, despite a lack of evidence for its effectiveness or safety for this purpose, is cause for concern. A third of young women believed N-tablet to be very effective in preventing pregnancy, and 15% had ever used it to this end, suggesting the need for informational campaigns regarding correct use of this medication. These findings support previous evidence on N-tablet’s use as a contraceptive in Ghana: in a survey of 220 N-tablet users, 94% (the majority aged 25 and under) had used it as pre-coital contraception and 88% did not know of any other uses for it; they perceived it to be effective and more convenient than taking daily contraceptive pills [18]. The perceived effectiveness of N-tablet as a contraceptive (and to a lesser extent abortifacient) likely derives from its known effect on regulating menses, which women may understand as having an action similar to EC. Based on this understanding, women who want a peri-coital contraceptive method that can be used as and when needed may favor N-tablet over EC, which is at least three times as expensive as N-tablet. For women looking to bring back late menses in case of suspected pregnancy, N-tablet may be chosen over the medical abortion combination of misoprostol and mifepristone, which is around 100 times more expensive than N-tablet.

### Social network influence

The majority of 15–24 year-olds were supportive of contraception and had supportive friends and partners (more so than older women). If partners did oppose contraception, it was reportedly predominantly for health (rather than moral) reasons. The associations between respondents’ support for contraception and friends and partners’ support is not surprising, and the causal pathway likely goes in both directions. However, many of these assessments of partners’ and friends’ opinions may rely on respondent assumptions, since only slightly over half of respondents reported discussing contraception with their partner and their friends.

Compared to older women, young women were more heavily influenced by their friends’ opinions. For young people who are unable to access health facilities and who may lack comprehensive sexuality education in school [50], friends may provide a key source of information on contraceptives. The strong influence of friends on young women’s opinions about contraception could potentially be leveraged through tools such as social media, where experts and influencers can disseminate accurate contraceptive information that is then picked up and shared by young people among their social networks. These channels present valuable opportunities to reach young people who may not be able to access or feel comfortable attending health facilities for contraceptive information.

While friends’ influence on respondents’ contraceptive use operated entirely through influencing their own opinions, partners’ opinions had a significant influence on respondents’ contraceptive use independently of respondents’ own opinions; partners’ opinions may shape decisions even when the two parties disagree. Interestingly, contraceptive use was higher when decisions were made jointly or by the partner (versus the respondent), unlike in a previous study [51]. This may be because the sample of respondents who reported being the main decision-makers included women who were not in stable unions where such discussions would more likely occur and where contraceptive use may be more likely in the first place.

This study has several strengths. The sample was nationally representative, and the survey covered numerous topics that have received little attention among young people to date (such as preferred contraceptive attributes and abortion practices), enabling us to help fill important knowledge gaps regarding reproductive needs of young Ghanaians. A major limitation is our moderate sample size of 15–24 year-olds, which limited our statistical power to detect differences in some analyses, and prevented us from looking at correlates of use of abortion. Another point to note is that our sample is restricted to young people who had ever had sex, who are not necessarily representative of the general population aged 15–24; therefore, our findings should not be generalized to all young people.

## Conclusions

This study highlights several important contraceptive and abortion needs specific to young people. Their desire to safeguard their fertility, along with patterns of more intermittent sex, may contribute to use of short-acting and coital-dependent methods such as EC, condoms and withdrawal. Since these methods have higher failure rates than longer-acting methods, young women predictably experience higher rates of unintended pregnancy, which they resolve more often with abortion. Yet the information young people receive on both contraception and abortion is inadequate. Young women’s perceptions of negative provider attitudes, poor knowledge of the abortion law, coupled with cost barriers, discourage many of them from attending facilities for contraceptive or abortion services, pushing them to resort to potentially unsafe or ineffective methods of abortion and contraception (such as N-tablet), and to source their information from friends who may also lack accurate knowledge. The Ministry of Health/Ghana Health Service, in partnership with youth-focused civil society organizations, can leverage the power of social media and broadly supportive social networks to develop strong public information campaigns to educate young people about their contraceptive and abortion options. School-based comprehensive sexuality education is another crucial avenue to build young people’s skills to make informed decisions about their sexual and reproductive lives. For those young women who are able to attend facilities, provider bias may reduce the quality of contraceptive counseling they receive, limiting their options for longer-acting methods and leading some to discontinue contraception altogether. Yet contraceptive counseling offers an opportunity to alleviate widespread concerns about certain methods causing permanent infertility, and to discuss potential side effects, health risks and alternative methods. Programs are needed to better train providers in implementing the National Operational Guidelines and Standards for Adolescent and Youth-friendly Health Services [36], to ensure they are offering comprehensive, confidential and non-discriminatory information and services that support young people in achieving their sexual and reproductive health and rights.

## Data Availability

The datasets used for the current study are available from the corresponding author on reasonable request.

## Appendix A: Weighted percent distribution of respondents who ever had sex by sociodemographic characteristics, for 15–24 and 25–49 year-olds

**Table:**

	15–24	25–49	
	(N = 1039)	(N = 3100)	p-value
*Residence*			0.00
Urban	51.1	60.7	
Rural	48.9	39.3	
*Zone*			0.00
Northern	14.5	13.1	
Middle	51.2	44.4	
Coastal	34.3	42.6	
*Union/marital status*			0.00
Married or cohabiting	47.8	73.1	
Formerly married or cohabiting	10.4	18.8	
Never married nor cohabiting	41.8	8.1	
*Sexual activity*			0.00
Had sex in last month	52.6	61.6	
Had sex > 1 month ago	47.4	38.4	
*Pregnancy/birth history*			0.00
Never pregnant	42.7	5.6	
Ever pregnant, no birth	13.2	4.2	
1 birth	26.4	14.5	
2 + births	17.7	75.6	
*Pregnancy status*			0.00
Not pregnant	83.0	76.5	
Trying to become pregnant	7.6	16.0	
Pregnant	9.4	7.5	
*Education*			0.00
None	5.5	19.1	
Primary	14.9	21.5	
Middle or higher	79.6	59.4	
*Wealth*			0.00
Poorest 60%	65.1	54.6	
Richest 40%	34.9	45.4	
*Importance of avoiding pregnancy*			0.00
Very important	75.6	57.5	
Somewhat important	6.7	8.7	
Not at all important	17.7	33.8	
*Contraceptive use*			0.00
Never used	43.6	37.4	
Previously used	24.5	32.3	
Currently using	31.9	30.4	
*Among ever pregnant respondents*			
*Intendedness of current pregnancy/last birth**			0.00
Wanted then	34.2	59.7	
Wanted later or not at all	65.8	40.3	

Sample is weighted to 2017 Ghana Maternal Health Survey, and excludes women who have never had sex

## Appendix B: Adjusted odds ratios of current contraceptive use according to indicators of opposition to contraception, among women aged 15–24

**Table:**

	Adjusted^a^ OR (95% CI)
*Respondent view on contraception*	
Support	(ref)
Oppose	0.37 (0.16, 0.84)*
Don't know	n/a
*Decision-maker on contraceptive use*	
Mainly respondent	(ref)
Mainly partner	4.22 (2.29, 7.77)*
Joint	87.87 (26.46, 291.77)*
Other	1.89 (0.47, 7.53)
*Partner's view on contraception*	
Oppose	0.49 (0.25, 0.98)*
Support	(ref)
Don't know	0.81 (0.42, 1.57)
*Proportion of friends who oppose contraception*	
Most	1.05 (0.55, 2.00)
About half	0.88 (0.43, 1.82)
Few	(ref)
None	1.41 (0.68, 2.95)

N = 826. Model excludes women who were pregnant or trying to get pregnant at the time of the survey

^a^Adjusted for other variables in table, as well as ecological zone, residence, union status, educational attainment and wealth

*p < 0.05

## Appendix C. Adjusted odds ratios of respondent opposing contraception according to social network opposition to contraception, among women aged 15–24

**Table:**

	Adjusted^a^ OR (95% CI)
*Partner's view on contraception*	
Opposes	13.17 (8.16, 21.23)*
Supports	(ref)
Respondent doesn't know	6.75 (3.82, 11.94)*
*Proportion of friends who oppose contraception*	
Most	1.89 (1.23, 2.91)*
About half	1.47 (0.75, 2.85)
Few	(ref)
None	1.17 (0.76, 1.81)

N = 1038

^a^Adjusted for other variables in table, as well as ecological zone, residence, union status, educational attainment and wealth

*p < 0.05

## Abbreviations

EC: Emergency contraception
FABM: Fertility awareness-based method
KNUST: Kwame Nkrumah University of Science and Technology
LARC: Long-acting reversible contraceptive

## Footnotes

1 We cite the most recent source of data for each indicator. For most indicators, this is the 2017 Maternal Health Survey. However, data on unintended births and unmet need were not available from the 2017 Maternal Health Survey; they were only available from the most recent Demographic and Health Survey (2014).

2 The World Health Organization defines an abortion as unsafe if it is conducted by persons lacking the necessary skills or in an environment lacking minimal medical standards or both.

3 Male sterilization was not reported by any respondents.

4 Following standard practice for abortion incidence rates, the denominator also includes women who have never had sex.

5 Formal providers comprise private doctors, midwives, hospitals, clinics (including mobile and family planning clinics), pharmacies, NGOs, CHPS and maternity homes.

6 Non-formal providers include traditional providers (traditional birth attendants and healers), fieldworkers, shops and markets, and social networks such as church, friends and relatives.
